# Near-infrared autofluorescence pattern in parathyroid gland adenoma

**DOI:** 10.1007/s00464-024-11314-8

**Published:** 2024-10-09

**Authors:** Leonardo Rossi, Andrea De Palma, Piermarco Papini, Malince Chicas Vasquez, Filomena Cetani, Carlo Enrico Ambrosini, Gabriele Materazzi

**Affiliations:** 1 Department of Surgical, Medical, and Molecular Pathology and Critical Area, University of Pisa, 56124 Pisa, Italy; 2 Endocrine Unit, University Hospital of Pisa, Pisa, Italy

**Keywords:** Parathyroid, Hyperparathyroidism, Near-infrared autofluorescence, NIRAF pattern, Parathyroid adenoma, Autofluorescence pattern

## Abstract

**Background:**

Parathyroid gland (PG) surgery is often challenging due to the small size and indistinct nature of these glands. The introduction of intraoperative near-infrared autofluorescence (NIRAF) has shown promise in localizing parathyroid tissue. However, the NIRAF features of parathyroid adenomas remain unclear. The aim of this study is to assess the NIRAF pattern of parathyroid adenomas.

**Methods:**

Patients who underwent surgery for primary hyperparathyroidism at the University Hospital of Pisa, Endocrine Surgery Unit, between December 2021 and February 2022 were enrolled in this study. Intraoperative NIRAF patterns of suspected parathyroid adenomas were evaluated, with particular attention given to the presence of a bright cap.

**Results:**

A retrospective study was conducted on 11 patients with primary hyperparathyroidism who underwent parathyroidectomy at our institution. Histopathological examination of the 15 resected specimens confirmed 14 parathyroid adenomas (12 chief cell parathyroid adenomas, 1 oxyphil cell parathyroid adenoma, and 1 mixed cell parathyroid adenoma) and one schwannoma. All adenomas exhibited a heterogeneous NIRAF pattern, distinct from the homogeneous pattern observed in the schwannoma. A bright cap was identified in 9 out of 14 (64.3%) parathyroid adenomas (all chief cell adenomas). On the contrary, all 9 macroscopically normal PGs identified during surgery presented an homogeneous pattern.

**Conclusion:**

Our findings support the integration of NIRAF into parathyroid surgical procedures. The heterogeneous NIRAF pattern observed in parathyroid adenomas, often accompanied by a bright cap, offers a promising intraoperative diagnostic tool to differentiate hyperfunctioning from normal parathyroid tissue. Larger-scale randomized trials are warranted to further validate these findings.

Primary hyperparathyroidism (PHPT) is a common endocrine disorder, affecting approximately 25–28 individuals per 100,000 [1]. Its incidence has risen in recent decades due to increased screening for serum calcium and parathyroid hormone levels [2]. PHPT is characterized by excessive parathyroid hormone (PTH) secretion, which may lead to elevated serum calcium levels. Moreover, this condition can adversely affect multiple organs, contributing to conditions such as osteoporosis, kidney stones, and chronic kidney disease [3].

The majority of PHPT cases (75–85%) are caused by a solitary adenoma. However, multiglandular disease or, in about 1% of cases, parathyroid carcinoma can also be the underlying cause [4]. Furthermore, familial syndromes, such as multiple endocrine neoplasia (MEN), may be associated with PHPT [2, 3, 5].

Accurate preoperative localization of abnormal parathyroid glands (PGs) is crucial for successful surgical management of PHPT [6]. Technetium-99 m sestamibi scintigraphy and cervical ultrasound are commonly employed as first-line imaging modalities. While effective, these methods have limitations in sensitivity and specificity [7]. Second-line imaging, including magnetic resonance imaging (MRI), choline positron emission tomography-computed tomography (PET-CT), and four-dimensional computed tomography (4D CT), may be considered when initial imaging is inconclusive or discordant [8].

Surgery represents the primary option to cure PHPT [9]. Despite advances in imaging, parathyroid surgery remains challenging, even for experienced surgeons. Intraoperative techniques have been developed to aid in the identification of parathyroid adenomas, but definitive visualization remains elusive, particularly in cases with equivocal preoperative imaging, and relies mainly on surgeon’s visual assessment and experience.

Parathyroid near-infrared autofluorescence (NIRAF), firstly introduced by Paras and colleagues in 2011, has emerged as a potential adjunct to traditional surgical techniques [1]. Initial studies have explored the use of NIRAF to differentiate parathyroid adenomas from normal tissue, but results have been inconsistent.

The aim of this study is to assess the NIRAF pattern of parathyroid adenomas in patients affected by PHPT undergoing parathyroid surgery. This study was approved by the Internal Research Board.

## Materials and methods

This retrospective pilot study was performed at the Endocrine Surgery Unit of the University Hospital of Pisa from December 2021 to February 2022. We included patients affected by PHPT and who underwent parathyroidectomy, either with conventional open surgery, minimally invasive video-assisted parathyroidectomy (MIVAP) or video-assisted thoracic surgery (VATS), with NIRAF assistance. Exclusion criteria were: secondary HPT, tertiary HPT, and age < 18 years old.

Preoperative work-up consisted of clinical examination, neck ultrasound (US), Technetium-99 m sestamibi scintigraphy or SPECT-CT, blood test examination, and further imaging exams (e.g. TC-scan) if required.

The data collected and analyzed included age, sex, indication for surgery, pre-operative imaging, preoperative serum calcium and PTH levels, number of PGs identified intra-operatively, duration of the operation (defined as the time between the skin incision and the skin suture), intra-operative quick PTH decrease, the NIRAF pattern of parathyroid adenomas, the NIRAF pattern of normal parathyroid glands, the presence of a NIRAF bright cap, the serum calcium level at postoperative day 1, the length of hospital stay, the occurrence of surgical complications, and the histology of the removed lesions.

### Surgical operation

All parathyroidectomies were performed by experienced endocrine surgeons (more than 100 procedures per year). Parathyroid surgery with cervical approach (either conventional parathyroidectomy and MIVAP) was performed according to our standard clinical practice. After midline incision and blunt dissection of the strap muscles, the thyroid lobe was gently retracted using small retractors. Careful attention was paid to avoid injury to blood vessels, the thyroid capsule, or the recurrent laryngeal nerve. For patients with preoperative imaging studies that concordantly identified adenomas, a targeted parathyroidectomy was performed. In cases of discordant or uncertain preoperative imaging, or for patients with suspected bilateral multiglandular disease, a bilateral neck exploration was conducted. Given the embryological origins of the parathyroid glands, the superior gland was initially sought posterior to the thyroid lobe, while the inferior gland was typically found in a more ventral position. However, due to the known anatomical variability of PGs location, sometimes further dissection was necessary.

Intraoperatively, once a suspected PG was identified, ambient lighting was turned off. The gland's NIRAF pattern was then evaluated using the Visionsense VS3 iridium system (Medtronic, Minneapolis, MN, USA) and classified as either “homogeneous” or “heterogeneous”. The presence of a bright cap was also assessed according to Demarchi et al. [10]. In case of identification of suspected normal PG, these were assessed in NIRAF mode and labelled according the same criteria. To ensure an accurate evaluation avoiding background or false-positive interference, each excised specimen was reinspected in NIRAF mode. All parathyroidectomies were guided by intraoperative quick PTH assays. Drainage was usually omitted except in specific cases or when indicated.

Regarding VATS procedure, it was performed using three trocar incisions in the left axillary triangle. The thymic remnant, suspected as the site of the parathyroid adenoma, was identified. The NIRAF pattern was then evaluated using the Visionsense VS3 iridium system (Medtronic, Minneapolis, MN, USA), which revealed a 2-cm round heterogeneous lesion. Both the thymic remnant and the lesion were excised and sent for frozen section, which confirmed the adenomatous nature of the PG. The procedure was guided by intraoperative quick PTH assays. Drainage tube placement.

## Results

Eleven patients affected by primary hyperparathyroidism (HP) who underwent parathyroidectomy were enrolled in the present study, including 10 women (90.9%) and 1 man (9.1%). Mean age was 62.8 ± 7.4 years, and mean BMI was 25.8 ± 6.5 kg/m^2^. Mean preoperative serum PTH level was 126.4 ± 58.8 pg/mL and mean preoperative serum calcium level was 11.5 ± 1.0 mg/dL, respectively. Ten patients had sporadic PHPT (90.0%), while one patient had suspected MEN 4-related PHPT (9.1%). Mean operative time was 42.7 ± 11 min. All patients were successfully treated, with postoperative serum calcium levels within the normal range in all cases. Mean postoperative serum calcium level was 9.4 ± 0.7 mg/dL. 10 out of 11 patients (90.9%) were discharged on postoperative day 1, while the patient (9.1%) who underwent VATS parathyroidectomy was discharged on postoperative day 4. A drainage tube was placed in two patients (18.2%): patient no. 9, who underwent VATS; and patient no. 10, who underwent a combined thyroidectomy and parathyroidectomy. We documented only 1 surgical complication (transient recurrent laryngeal nerve palsy spontaneously recovered; patient no. 1). Overall, 15 neck masses were removed: histological examination confirmed 14 parathyroid adenomas (93.3%) and 1 schwannoma (patient no. 8) (6.7%). The parathyroid adenomas were classified as follows: 12 chief cell parathyroid adenomas (85.7%), 1 oxyphil cell parathyroid adenoma (7.1%), and 1 mixed cell parathyroid adenoma (7.1%). These data are summarize in Table 1.

**Table 1:** Clinical, demographics, surgical and histological data

Age (years; mean ± SD)	62.8 ± 7.4
BMI (Kg/m^2^; mean ± SD)	25.8 ± 6.5
Sex distribution	
Female (n;%)	10 90.9%)
Male (n;%)	1 (9.1%)
Preoperative serum PTH (pg/mL; mean ± SD)	126.4 ± 58.8
Preoperative serum calcium level (mg/dL; mean ± SD)	11.5 ± 1
Mean operative Time (min; mean ± SD)	42.7 ± 11
Postoperative serum calcium level (mg/dL; mean ± SD)	9.4 ± 0.7
Successful operations (n; %)	11 (100%)
Median post-operative length of hospital stay (days; median range)	1 (1–4)
Mean parathyroid adenoma size (mm; mean ± SD)	18.1 ± 4.7
Histology (n; %)	
Chief cell parathyroid adenoma	12 (80.0%)
Oxyphil cell parathyroid adenoma	1 (6.7%)
Mixed cell parathyroid adenoma	1 (6.7%)
Schwannoma	1 (6.7%)

*BMI* body mass index, *SD* standard deviation, *PTH* Parathyroid hormone

Pre-operative imaging concordance was documented in 9 out of 11 patients (81.8%) for 13 out of 14 parathyroid adenomas (92.9%). Specifically, 6 parathyroid adenomas were identified by both US and SPECT-CT, 2 by US and Technetium-99 m sestamibi scintigraphy, 1 by US, Technetium-99 m sestamibi scintigraphy, and CT-scan, 3 by US and CT-scan, and 1 mediastinal parathyroid adenoma was identified by SPECT-CT and CT-scan. Notably, pre-operative imaging for patient no. 8 indicated a suspected left inferior parathyroid adenoma and a 18 × 12 mm right central compartment neck mass without Technetium-99 m sestamibi scintigraphy uptake. Moreover, the imaging concordance was not assessed for patient no. 10 since the parathyroid adenoma was documented at the pre-operative US but no Technetium-99 m sestamibi scintigraphy or SPECT-TC were performed. Targeted single parathyroidectomy was performed in 7 cases (63.6%) (5 via conventional open surgery, 1 via VATS, and 1 via MIVAP), while in another case (patient no. 3) (9.1%) two targeted parathyroidectomies were performed. Furthermore, in one case (patient no. 2) (9.1%), a bilateral neck exploration was performed and 3 out of 4 PGs were removed due to suspected MEN 4 syndrome. Moreover, in one case (patient no. 10) (9.1%), targeted parathyroidectomy was performed along with total thyroidectomy due to a multinodular goiter. Lastly, in one case (patient no. 8) (9.1%) a bilateral neck exploration was performed and one PG was removed along with a right central compartment mass. Overall, 14 out of 23 identified (60.9%) PGs were removed. A detailed description of each surgical case is reported in Table 2.

**Table 2:** Patient baseline and clinical features, preoperative imaging, surgical data

Patient no	Sex	Age	BMI	Diagnosis	US	Sestamibi SCINTIGRAPHY	SPECT -TC	TC	Imaging localization concordance	Surgical operation	Number of PG identified	Resected gland	Bilateral exploration	Operative time (min)	Successful i.o. PTH assay
1	F	52	22	sPHPT	R S	NP	R S	NP	Yes	Target Parathyroidectomy	2	RS	No	40	Yes
2	M	66	32	MEN 4	R S	NP	R S	NP	Yes	Neck Exploration, Multiple parathyroidectomy	4	RS	Yes	45	Yes
					R I	NP	R I	NP	Yes			RI			
					L S	NP	L S	NP	Yes			LS			
3	F	70	37	sPHPT	L S	NP	NP	L S	Yes	Multiple Target Parathyroidectomy	2	LS	No	55	Yes
					L I	NP	NP	L I	Yes			LI			
4	F	63	37	sPHPT	R I	NP	R I	NP	Yes	Target Parathyroidectomy	2	RI	No	50	Yes
5	F	56	18	sPHPT	L I	NP	L I	L I	Yes	Target Parathyroidectomy	2	LI	No	30	Yes
6	F	75	25	sPHPT	L I	NP	L I	NP	Yes	Target Parathyroidectomy	1	LI	No	40	Yes
7	F	58	23	sPHPT	R S	R S	NP	NP	Yes	Target Parathyroidectomy	1	RS	No	25	Yes
8	F	68	22	sPHPT	L I	L I	NP	L I	Yes	Neck exploration, Left inferior Parathyroidectomy + Right Central Compartment Mass removal	3	LI	Yes	50	Yes
					R S	Neg	NP	R S	No			RCCM			
9	F	65	22	sPHPT	Neg	NP	MEDIASTINUM	MEDIASTINUM	Yes	Video-Assisted Thoracic Target Parathyroidectomy	1	Mediastinal PG	No	60	Yes
10	F	52	25	sPHPT + MNG	R I	NP	NP	NP	NA	Target Parathyroidectomy + Total Thyroidectomy	3	RI	No	45	Yes
11	F	66	21	sPHPT	L S	L S	NP	NP	Yes	Minimally Invasive Video-Assisted Target Parathyroidectomy	2	LS	No	30	Yes

*BMI* body mass index, *US* ultrasound, *I.O*. intra-operative, *PTH* parathyroid hormone, *sPHPT* sporadic primary hyperparathyroidism, *MNG* multinodular goiter, *L* left, *R* right, *S* superior, *I* inferior, *NP* not performed, *NA* not available, *RCCM* right central compartment mass, *PG* parathyroid gland

**Figs. 1 and 2:**
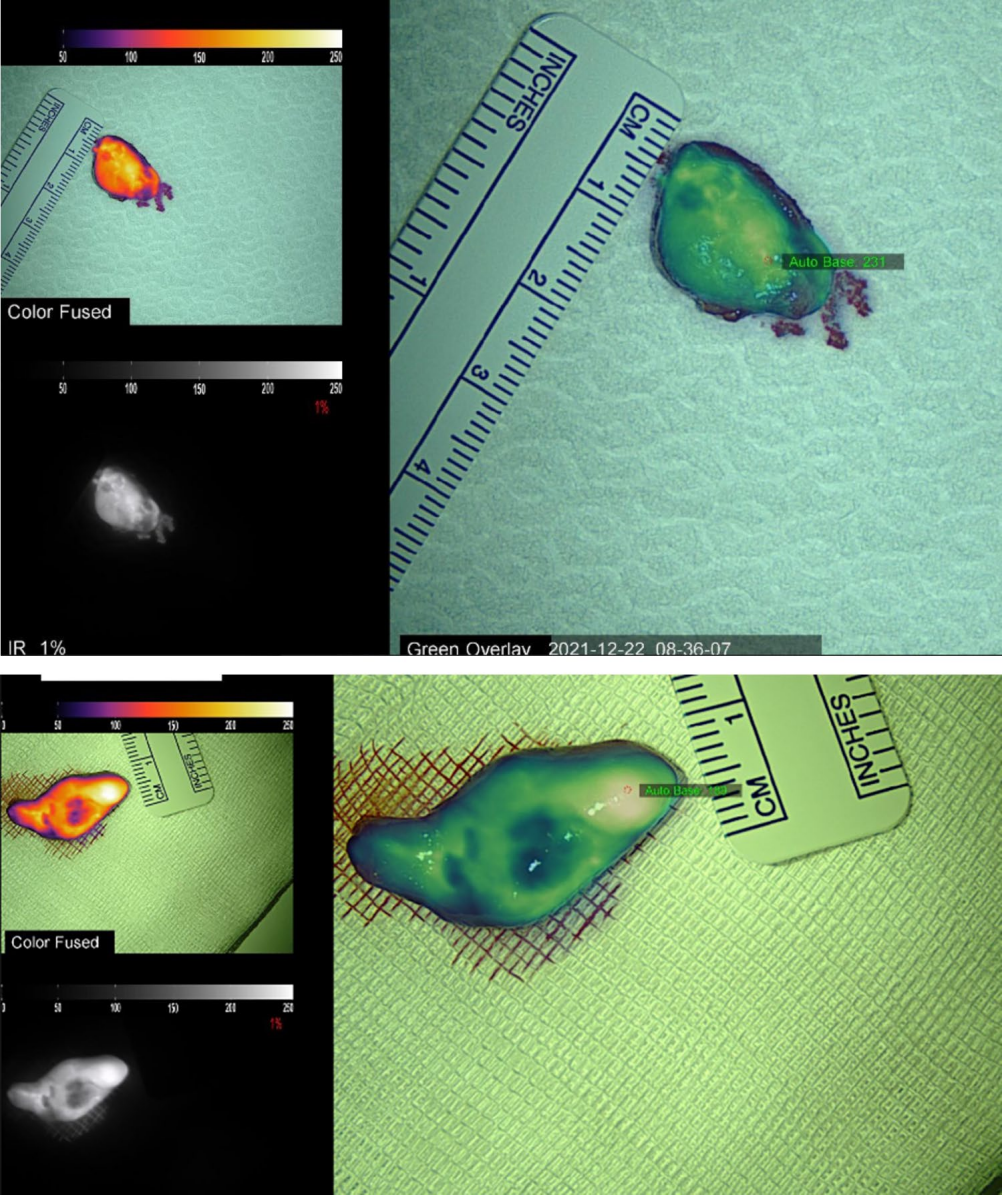
Parathyroid gland adenoma heterogenous NIRAF pattern

**Fig. 3:**
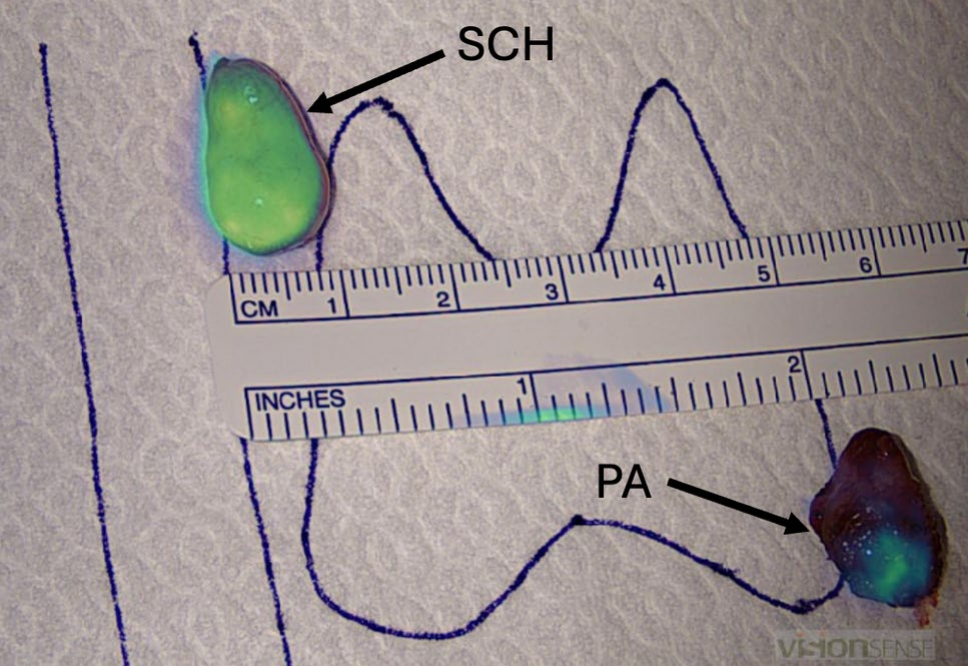
Different NIRAF patterns between schwannoma (homogeneous) and parathyroid adenoma (heterogeneous). *SCH* schwannoma, *PA* parathyroid adenoma (see arrows)

**Fig. 4:**
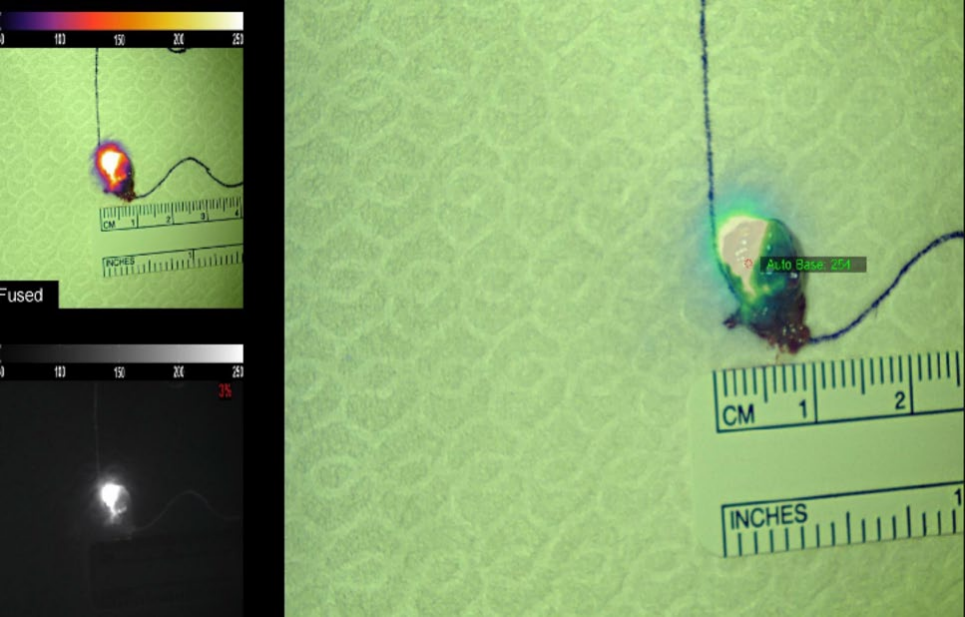
Parathyroid gland adenoma shows an heterogenous pattern with a well circumscribed bright cap

**Fig. 5:**
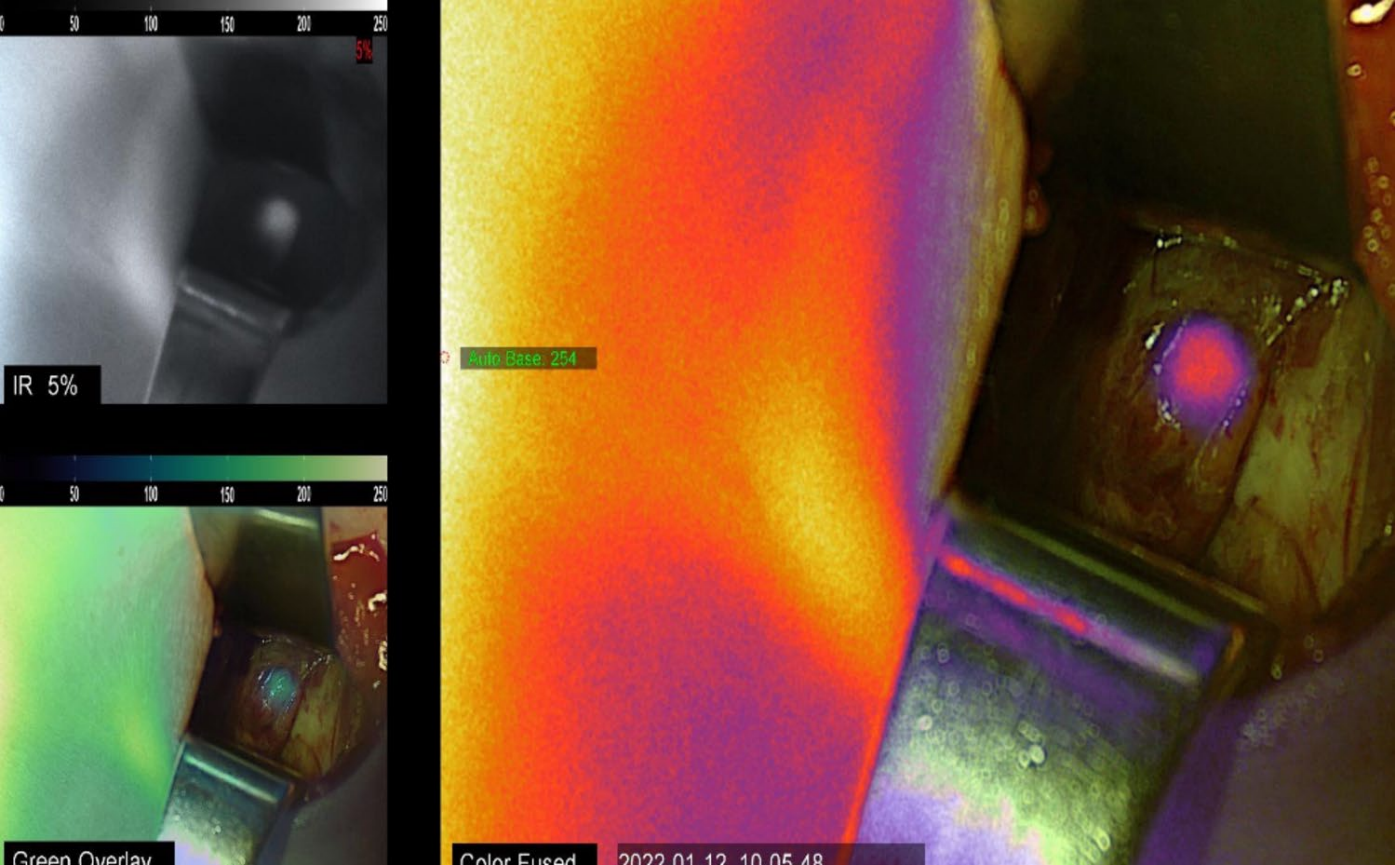
Intraoperative NIRAF pattern of normal parathyroid gland with a well-defined homogeneous NIRAF pattern

NIRAF revealed a heterogeneous pattern in all parathyroid adenomas (Figs. 1 and 2), whereas the schwannoma showed homogeneous enhancement (Fig. 3). Furthermore, in 9 out of 14 parathyroid adenomas (64.3%) (all chief cell adenomas) a bright cap was clearly identified (Fig. 4), while it was absent in 5 cases (35.7%) (3 chief cell parathyroid adenomas, 1 oxyphil cell parathyroid adenoma, and 1 mixed cell parathyroid adenoma). All 9 macroscopically normal PGs identified presented an homogeneous pattern (Fig. 5). NIRAF features of the study population are reported in Table 3.

**Table 3:** Histological and NIRAF Data

Patient no	Resected gland	Histology	NIRAF Pattern	Bright cap
1	R S	Chief cell parathyroid adenoma	Heterogenous	No
2	R S	Chief cell parathyroid adenoma	Heterogenous	Yes
	R I	Oxyphil cell parathyroid adenoma	Heterogenous	No
	L S	Chief cell parathyroid adenoma	Heterogenous	Yes
3	L S	Chief cell parathyroid adenomas	Heterogenous	Yes
	L I	Chief cell parathyroid adenomas	Heterogenous	Yes
4	R I	Chief cell parathyroid adenomas	Heterogenous	Yes
5	L I	Chief cell parathyroid adenomas	Heterogenous	Yes
6	L I	Chief cell parathyroid adenomas	Heterogenous	Yes
7	R S	Chief cell parathyroid adenomas	Heterogenous	Yes
8	L I	Chief cell parathyroid adenomas	Heterogenous	Yes
	RCCM	Schwannoma	Homogenous	No
9	MEDIASTINAL PG	Chief cell parathyroid adenomas	Heterogenous	No
10	R I	Chief cell parathyroid adenomas	Heterogenous	No
11	L S	Mixed cell parathyroid adenoma	Heterogenous	No

*R* right, *L* left, *S* superior, *I* inferior, *RCCM* right central compartment mass, *NIRAF* near infrared autofluorescence

## Discussion

Surgery for hyperparathyroidism remains challenging, especially when preoperative imaging is inconclusive. The primary difficulty lies in accurately differentiating adenomatous parathyroid glands from normal ones intraoperatively. This study aimed to explore the potential of NIRAF as an intraoperative tool to improve the accuracy of PG identification and discrimination. To our knowledge, this is the first study to analyze NIRAF data using the Visionsense VS3 Iridium System® (Medtronic, Minneapolis, MN, USA).

NIRAF has emerged as a promising adjunct in neck surgery since its introduction by Paras et al. in 2011 [1]. The authors reported significantly elevated fluorescence intensity within PGs compared to adjacent neck tissues when stimulated by 785 nm near-infrared light, with subsequent emission centered around 820–830 nm. This enhanced autofluorescence has facilitated intraoperative parathyroid gland visualization, leading to improved preservation and subsequent parathyroid function following thyroidectomy [11–13].

Although the application of NIRAF in thyroid surgery has garnered considerable interest in the scientific community, its use in hyperparathyroidism surgery is limited to a few studies with a small number of patients. Our study revealed a heterogeneous NIRAF pattern in all parathyroid adenomas, in contrast to the homogeneous signal in normal parathyroid glands. These findings align with Kose et al., who reported a low-intensity and heterogeneous NIRAF pattern in hyperfunctioning parathyroid glands, attributing this to histological changes such as increased cellularity, fibrosis, oxyphil cell clusters, and hematomas [14]. Interestingly, in our study a distinct bright cap was observed in 64.3% of parathyroid adenomas. This finding is consistent with Demarchi et al., who suggested that such a cap represents a rim of normal parathyroid tissue within the adenoma [10]. Nonetheless, the inconsistent presence of the bright cap, observed in only about 60% of cases, raises concerns about its reliability as a standalone marker for identifying parathyroid adenomas, warranting further investigation.

Moreover, Squires et al. demonstrated that NIRAF significantly increased the confidence in intraoperative parathyroid identification during parathyroidectomy, aiding in the identification of parathyroid tissue in 20% of cases and excluding other soft tissues in 15% [15]. However, contrasting results have been reported by Ladurner et al., who found no significant differences in NIRAF patterns between adenomas and normal PGs [16].

Interestingly, Berber et al. investigated whether distinct NIRAF patterns exist among various types of hyperparathyroidism, concluding that NIRAF patterns are remarkably consistent across different etiologies of PHPT, while in case of tertiary hyperparathyroidism parathyroid adenomas often exhibits a more homogeneous pattern [17]. Our study corroborates these findings, as we observed a heterogeneous NIRAF pattern in all cases of sporadic PHPT as well as in the single case of MEN4-related PHPT, in which all 3 resected adenomas showed an heterogenous pattern.

The definitive decision regarding which PG to excise during hyperparathyroidism surgery remained contingent upon the surgeon's intraoperative assessment, informed by preoperative data and real-time intraoperative PTH measurements. Pre-operative imaging remains the gold standard technique for guiding parathyroid surgery. Nonetheless, there are instances when pre-operative imaging may be inconclusive or intra-operative findings may raise doubts about the nature of a PG, particularly if it is small-sized. In this context, NIRAF may serve as an adjunctive tool to guide surgeons during hyperparathyroidism surgery, rather than replacing pre-operative imaging. We hypothesize that NIRAF may be particularly beneficial for surgeons with limited experience in visualizing and identifying abnormal PGs, as it can provide an additional visual information, thereby minimizing tissue dissection and reducing the risk of complications, such as recurrent laryngeal nerve injury and bleeding.

While NIRAF imaging offers a valuable adjunct for parathyroid localization, its tissue penetration depth is limited, often necessitating further surgical dissection for definitive identification [12]. The efficacy of NIR in detecting rare, intrathyroidal parathyroid adenomas remains uncertain and warrants further investigation.

While our findings suggest that NIRAF may be a promising tool for parathyroid surgery, the study harbors several limitations. As a single-center study with a relatively small sample size, the generalizability of our results may be limited. Furthermore, the subjective interpretation of NIRAF patterns and the lack of standardized quantitative measures hinder reproducibility. The absence of a control group precludes definitive conclusions regarding the specificity and sensitivity of NIRAF. Future studies should address these limitations by incorporating larger, multicenter cohorts and developing standardized protocols for NIRAF image analysis.
